# Hematoma-driven immuno-epigenetic remodeling after intracerebral hemorrhage: cell-type-specific mechanisms and therapeutic opportunities

**DOI:** 10.3389/fimmu.2026.1895546

**Published:** 2026-08-28

**Authors:** Yifan Li, Wei Jiang, Fangtian Zhong, Ming Dong

**Affiliations:** Department of Neurology and Neuroscience Center, The First Hospital of Jilin University, Changchun, China

**Keywords:** blood–brain barrier, immuno-epigenetic remodeling, intracerebral hemorrhage, neuroinflammation, precision therapeutics, microglia/macrophages

## Abstract

Intracerebral hemorrhage (ICH) is a life-threatening subtype of stroke characterized by the acute accumulation of blood within the brain parenchyma and progressive secondary brain injury. After ICH, dysregulated neuroinflammation drives a cascade of secondary injury processes that shape neurological deterioration and long-term recovery. However, the molecular mechanisms that determine the magnitude, temporal evolution, and resolution of immune-inflammatory responses after ICH remain incompletely understood, limiting the development of targeted therapeutic strategies. Accumulating evidence indicates that epigenetic regulation constitutes a critical layer controlling neuroinflammatory programs after ICH. Hematoma-derived stimuli, including hemoglobin degradation products, iron overload, oxidative stress, and damage-associated molecular patterns, create a unique inflammatory microenvironment that reshapes gene-regulatory landscapes in resident and infiltrating cells. DNA methylation remodeling, histone modification dynamics, chromatin accessibility alterations, and non-coding RNA regulatory networks collectively orchestrate cell-type–specific transcriptional reprogramming in microglia, astrocytes, endothelial cells, neurons, and infiltrating leukocytes. Rather than serving as passive consequences of tissue injury, these epigenetic processes actively modulate innate immune activation, cytokine production, leukocyte recruitment, blood–brain barrier integrity, and the balance between neurotoxic and reparative inflammatory states. In this review, we synthesize current evidence on the immuno-epigenetic regulation of neuroinflammation after ICH, with emphasis on cell-specific mechanisms, temporal dynamics, and immune–vascular interactions. We discuss how epigenetic reprogramming contributes to inflammatory amplification, glial phenotypic transitions, endothelial dysfunction, and the potential persistence of maladaptive inflammatory memory. Finally, we highlight emerging precision strategies, including locus-specific epigenome editing and RNA-based therapeutics, that may enable targeted modulation of neuroinflammation after ICH. An immuno-epigenetic perspective may provide a conceptual framework for developing precision neurotherapeutics for ICH.

## 1 Introduction

Spontaneous ICH is defined as the non-traumatic rupture of cerebral vasculature, resulting in extravasation of blood into the brain parenchyma (1). Although ICH is less prevalent than ischemic stroke, it accounts for a disproportionately high burden of mortality and long-term functional disability (2). Hematoma expansion contributes to early neurological deterioration, but secondary brain injury is further sustained by hematoma-derived blood components that persist within the brain parenchyma. These extravasated blood components disrupt neurovascular homeostasis and trigger innate immune activation. Hematoma-derived blood products and damage-associated molecular patterns (DAMPs) are released from lysed erythrocytes and damaged neural cells. These molecules, including hemoglobin, heme, free iron, high-mobility group box 1 (HMGB1), and adenosine triphosphate (ATP), act as potent initiators of sterile neuroinflammation (3, 4). Resident microglia serve as the primary immune sentinels in the cerebral parenchyma. They mount one of the earliest immune responses to hematoma-derived DAMPs. This activation occurs through pattern recognition receptors, including TLR2, TLR4, and RAGE. Receptor engagement triggers rapid activation of NF-κB and MAPK signaling cascades. These pathways drive transcriptional upregulation of pro-inflammatory cytokines (e.g., TNF-α, IL-1β, IL-6) and chemokines (e.g., CCL2, CXCL1), which promote endothelial activation and blood–brain barrier disruption. This vascular breach facilitates the infiltration of neutrophils, inflammatory monocytes, and lymphocytes into the perihematomal region (5–7).

**Figure 1:**
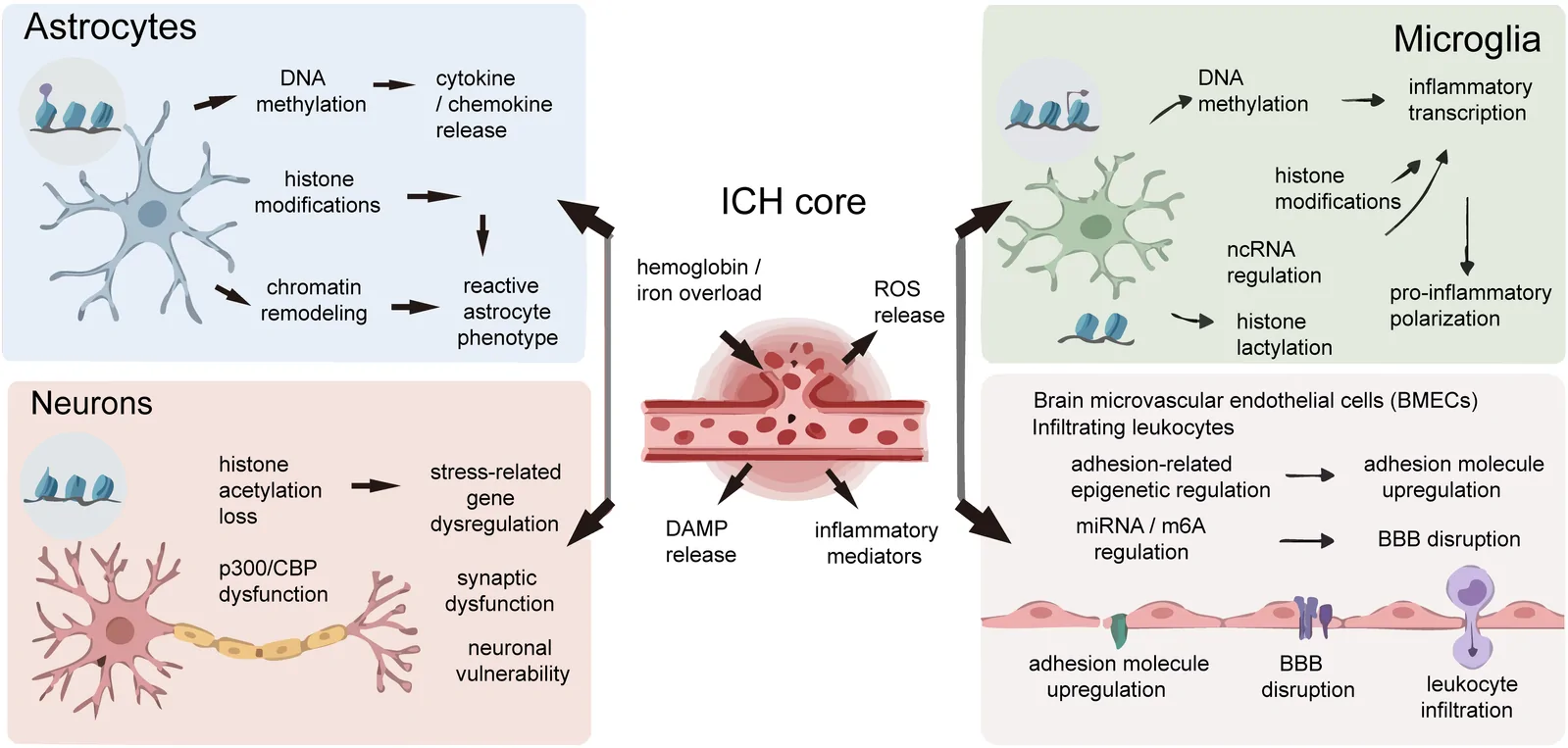
Cell-type-specific epigenetic regulation of neuroinflammation after intracerebral hemorrhage. After ICH, hematoma formation and red blood cell lysis lead to hemoglobin/iron overload, reactive oxygen species (ROS) production, damage-associated molecular pattern (DAMP) release, and the generation of inflammatory mediators within the hemorrhagic core. These injury-associated signals trigger epigenetic remodeling in multiple central nervous system cell types. In astrocytes, DNA methylation, histone modifications, and chromatin remodeling contribute to cytokine/chemokine release and the acquisition of a reactive astrocyte phenotype. In microglia, DNA methylation, histone modifications, non-coding RNA (ncRNA) regulation, and histone lactylation shape inflammatory and repair-associated transcriptional programs. In neurons, histone acetylation loss and p300/CBP dysfunction are associated with stress-related gene dysregulation, synaptic dysfunction, and neuronal vulnerability. In brain microvascular endothelial cells (BMECs), miRNA- and m6A-mediated regulation affects adhesion molecule expression, barrier integrity, and leukocyte infiltration. Collectively, these cell-type-specific epigenetic alterations amplify neuroinflammation, promote peripheral immune recruitment, exacerbate BBB dysfunction and neuronal injury, and ultimately contribute to secondary brain damage after ICH.

Acute inflammation worsens early tissue injury after ICH. Over time, the inflammatory response shifts from hematoma-derived toxicity, blood-brain barrier (BBB) disruption, and innate immune activation toward hematoma clearance, glial remodeling, vascular repair, and resolution-associated immune programs. TREM2-positive microglia, Tregs, and pro-resolving or phagocytic macrophage programs become more prominent during these later stages and contribute to hematoma resolution, myelin debris clearance, and extracellular matrix remodeling (8). These processes overlap rather than occur as discrete steps, and their timing may vary according to hematoma volume, location, and systemic inflammatory status. Thus, neuroinflammation after ICH is not a static destructive process, but a temporally evolving cascade whose trajectory must be tightly regulated. Notably, the molecular regulators governing the intensity, spatial distribution, and dynamic evolution of this neuroinflammation remain incompletely characterized. Epigenetic regulation provides a dynamic and context-dependent layer that shapes inflammatory gene expression after ICH. This regulatory stratum encompasses DNA methylation, histone post-translational modifications, ATP-dependent chromatin rearrangement, and non-coding RNA-mediated networks. Therefore, this review synthesizes current evidence on how epigenetic remodeling regulates neuroinflammation after ICH and discusses its therapeutic implications. **Figure 1** provides a schematic overview of the major cell-type-specific epigenetic mechanisms involved in neuroinflammation after ICH.

## 2 Epigenetic remodeling of neuroinflammation

After hematoma-induced stress, inflammatory gene expression is not governed solely by upstream cytokine signaling. DNA methylation, histone modifications, and non-coding RNAs also help determine which inflammatory programs are activated, sustained, or resolved over time. Time-resolved snATAC-seq has shown that chromatin accessibility changes across cell types and injury stages after ICH (9).

### 2.1 Dynamic remodeling of DNA methylation

DNA methylation is a major epigenetic mechanism regulating inflammatory gene expression. DNA methylation is regulated by DNA methyltransferases (DNMTs) and ten-eleven translocation (TET) dioxygenases. Methyl-CpG-binding proteins (MBPs) are responsible for interpreting methylated DNA.

DNMTs catalyze the transfer of a methyl group to the C5 position of cytosine residues, generating 5-methylcytosine (5mC). DNA methylation at promoter-associated CpG sites is generally linked to transcriptional repression (10). TET dioxygenases successively oxidize 5mC to 5-hydroxymethylcytosine (5hmC), 5-formylcytosine (5fC), and 5-carboxylcytosine (5caC), thereby initiating pathways of DNA demethylation (11). Evidence from microglial inflammatory models shows that TET2 is induced by inflammatory stimuli and regulates early transcriptional and later pro-inflammatory responses (12). These findings suggest that TET-dependent mechanisms may contribute to inflammatory gene regulation after ICH, although direct ICH-specific evidence remains limited. In a tumor endothelial model, ICAM-1 silencing was associated with promoter histone H3 deacetylation and loss of H3K4 methylation, rather than DNA hypermethylation (13). Direct evidence that promoter demethylation of ICAM-1 or VCAM-1 regulates leukocyte transmigration after ICH is lacking. Overall, current evidence is more consistent with locus- and cell-specific regulation than with a uniform genome-wide increase or decrease in DNA methylation after ICH.

### 2.2 Dynamic regulation of histone modifications

Histone modifications critically regulate the chromatin accessibility of inflammatory genes after ICH, thereby shaping the neuroinflammatory response. These modifications involve the site-specific addition or removal of covalent chemical groups, such as acetyl and methyl groups, on histone tails. Histone proteins are subject to a diverse array of covalent post-translational modifications, prominently including lysine acetylation and arginine or lysine methylation. These marks remodel chromatin structure and histone–DNA interactions, thereby influencing the recruitment of transcriptional regulators to specific genomic loci (14). Histone acetyltransferases (HATs) promote histone acetylation and are generally linked to active transcription. Histone deacetylases (HDACs) remove acetyl groups from histones and often suppress gene expression (15). Histone methylation can have activating or repressive effects on gene expression, depending on the modified lysine or arginine residue and the degree of methylation (16). H3K4me3, a histone mark enriched at transcriptionally active promoters, has been implicated in inflammatory transcription after ICH. In an ICH-related microglial model, TBX21-mediated repression of SIRT1 enhanced WDR5-associated H3K4me3 and increased the production of pro-inflammatory mediators, including IL-1β, IL-6, and TNF-α (17). Moreover, histone modifications influence microglial state transitions, contribute to BBB disruption, and sustain chronic neuroinflammation after ICH. Genetic or pharmacological perturbation of key histone-modifying axes, exemplified by the TBX21–SIRT1–WDR5–H3K4me3 pathway, attenuates neuroinflammation and improves functional recovery in preclinical ICH models (17). The lactate–p300/CBP–H3K18la axis in microglia promoted white matter repair after ICH, whereas inhibition of this pathway aggravated white matter injury (18). This reinforces the functional relevance of epigenetic regulation in resolving inflammation after ICH. Histone modifications can influence both inflammatory activation and repair after ICH. Their effects vary according to the modified residue, cell type, and injury stage.

### 2.3 The regulatory network of non-coding RNA

ncRNAs are a diverse class of functional RNA transcripts that do not encode proteins but instead exert precise control over gene expression. They primarily function at the transcriptional and post-transcriptional levels through sequence-specific interactions with nucleic acids and proteins. ncRNAs are abundantly and differentially expressed in astrocytes and microglia, and accumulating evidence supports their functional role as key mediators of neuroinflammatory processes (19). As the most extensively characterized subclass of ncRNAs, miRNAs are endogenous small non-coding molecules (~18–25 nt) that post-transcriptionally regulate hundreds of target genes through sequence-specific base pairing with mRNAs (20). miRNAs modulate glial-mediated neuroinflammation by regulating microglial state transitions, astrocyte reactivity, and cell survival after ICH. The effects of miRNAs after ICH are context dependent and may change over the course of injury. Different miRNAs have been implicated in inflammatory amplification, anti-inflammatory signaling, and repair-related responses. However, their temporal and cell-specific patterns after ICH remain incompletely characterized (21). For example, certain miRNAs function as pro-inflammatory mediators during the acute phase of ICH. Notably, miR-155 is rapidly upregulated in activated microglia and directly targets the 3′ untranslated region (3′ UTR) of SOCS1 mRNA, leading to translational repression and reduced SOCS1 protein expression. This attenuation of SOCS1-mediated negative feedback potentiates STAT1 signaling and amplifies downstream pro-inflammatory responses (22). In an ICH model, miR-124 shifted microglia toward a less inflammatory state and reduced brain inflammation through the C/EBP-α pathway (23). A related experimental autoimmune encephalomyelitis (EAE) study showed that miR-124 promoted microglial quiescence and suppressed macrophage activation through the C/EBP-α–PU.1 pathway (24). Some lncRNAs interact with chromatin-associated proteins and epigenetic regulators, thereby influencing chromatin structure and transcription (25). In ICH models, several lncRNAs have been linked to cell-specific inflammatory or injury responses, but direct evidence connecting these effects to chromatin-based regulation remains limited (26, 27). miRNAs and lncRNAs contribute to inflammatory and repair-related gene regulation after ICH.

## 3 Cell-type-specific epigenetic regulation after ICH

Representative epigenetic mechanisms in major cell types involved in neuroinflammation after ICH are summarized in **Table 1**.

**Table 1:** Representative epigenetic mechanisms and evidence sources in major cell types involved in neuroinflammation after ICH.

Cell type	Core epigenetic mechanisms	Representative axes	Evidence model	Functional impact
Astrocytes	DNA methylation; histone modifications; chromatin remodeling	Reactive astrocyte-associated chromatin programs; injury-stage-dependent astrocyte state transitions	ICH snATAC-seq; related neuroinflammatory models; partly extrapolated astrocyte evidence	Astrocyte activation; cytokine production; perihematomal inflammation
Microglia	Histone methylation/lactylation; DNA methylation; ncRNA regulation	TBX21–SIRT1–WDR5-H3K4me3; lactate–p300/CBP–H3K18la	*In vivo* ICH; microglial inflammatory/repair models	Microglial state transitions; inflammatory transcription; white matter repair
Neurons	Histone acetylation (CBP/p300); global deacetylation	CBP/p300-associated histone acetylation changes; H3K14 lactylation-related neuronal ferroptosis; HMGB1-related inflammatory signaling	*In vivo* ICH; neuronal injury models; stroke-related evidence	Neuronal vulnerability; synaptic dysfunction; secondary neuroinflammation
Endothelial cells	DNA methylation; ncRNA regulation; epitranscriptomic regulation	TET2-ZO-1 regulation in a related endothelial model; miR-126-3p/VCAM-1; miR-27a-3p/AQP11; METTL3–YTHDF1-BCL-3 m6A-associated ferroptosis	*In vivo* ICH; brain endothelial cell models; related vascular inflammatory models	Endothelial adhesion; barrier dysfunction; leukocyte transmigration; BBB-associated inflammation
Infiltrating immune cells	DNA methylation; histone modifications; ncRNA regulation	CHIP-associated DNMT3A/TET2/ASXL1 mutations; trained immunity-like myeloid chromatin remodeling; epigenetic control of regulatory T cell programs	Human vascular/immune studies; related stroke/neuroinflammatory models; limited ICH-specific evidence	Peripheral immune recruitment; inflammatory amplification; hematoma clearance; resolution-associated immune responses

ICH, intracerebral hemorrhage; BBB, blood–brain barrier; ncRNA, non-coding RNA; HMGB1, high-mobility group box 1; CHIP, clonal hematopoiesis of indeterminate potential.

Evidence models distinguish ICH-specific findings from related stroke, neuroinflammatory, vascular, or immune studies.

### 3.1 Epigenetic regulation in astrocytes

Astrocytes are essential regulators of central nervous system homeostasis and actively interact with neurons, endothelial cells, and immune cells under both physiological and pathological conditions (28, 29). Astrocytes exhibit a dual role in neuroinflammation: they can limit the infiltration of peripheral immune cells into the CNS parenchyma under pathological conditions yet simultaneously possess potent pro-inflammatory capabilities—particularly upon activation. Therefore, astrocytes are increasingly recognized as pivotal regulators of neuroinflammatory processes within the central nervous system (30). Substantial evidence indicates that epigenetic mechanisms contribute to this process, effectively regulating astrocyte activation, cytokine production, and related inflammatory responses. Potential epigenetic mechanisms involved in astrocyte reprogramming include chromatin accessibility remodeling, histone modification dynamics, and ncRNA-mediated regulation of inflammatory transcriptional programs. Prior work has identified DNA methylation, histone modifications, and miRNA networks as regulators of astrocyte function in neuroinflammatory conditions (31). More recent studies have linked MAFG/MAT2α-associated DNA methylation and ACLY/p300-dependent histone acetylation to inflammatory astrocyte programs (32, 33). Most of these mechanisms have been defined in related neuroinflammatory models rather than in the acute hematoma environment. Studies in EAE models have provided important insights into inflammatory astrocyte states and their interactions with resident and infiltrating immune cells (34). However, EAE is a chronic immune-mediated model and differs from the acute sterile injury environment of ICH. Recent time-resolved snATAC-seq analysis provided ICH-specific evidence of dynamic astrocyte chromatin remodeling. Xiang et al. profiled mouse brain nuclei at 1, 3, 7, 14, and 28 days after ICH. During the early phase, increased accessibility at candidate regulatory elements linked to *Tgm1* was associated with a protective astrocyte program. At later stages, accessibility increased around *Serping1* and *C3*, consistent with a shift toward neurotoxic reactivity. These states were inferred from chromatin accessibility and marker-associated regulatory profiles and still require astrocyte-specific functional validation (9). Despite this study, direct epigenetic evidence in ICH astrocytes remains limited. Further cell-type-resolved studies with causal perturbation are needed to identify regulators of astrocyte-driven inflammation and repair.

### 3.2 Epigenetic regulation in microglia

Microglia are the intrinsic immune sentinels of the brain, serving as resident macrophages of the CNS and representing a functionally distinct glial lineage (35). Microglia communicate bidirectionally with astrocytes and can jointly shape neuroinflammatory responses (36, 37). Microglia exhibit context-dependent phenotypic plasticity and occupy temporally evolving, functionally heterogeneous states after CNS injury. Rather than fitting into a rigid M1/M2 dichotomy, these states include inflammatory, phagocytic, and repair-associated programs that change across injury stages (38). Multiple epigenetic layers shape these microglial state transitions, including DNA methylation, histone post-translational modifications, and ncRNA pathways. These mechanisms can influence inflammatory and repair-associated transcriptional programs. Previous studies on microglial epigenetics suggest that these regulatory layers may stabilize inflammatory transcriptional states or facilitate transitions toward reparative programs. In this context, epigenetic remodeling may not only initiate microglial activation after ICH but also influence the reversibility and temporal persistence of these state changes (39, 40). Recent mechanistic work identified the TBX21–SIRT1–WDR5–H3K4me3 axis as a regulator of microglial inflammation after ICH. In OGD-treated BV2 microglia, TBX21 bound to the SIRT1 promoter and suppressed SIRT1 transcription. TBX21 knockdown increased SIRT1 expression and reduced WDR5 and H3K4me3 levels. It also decreased COX2, iNOS, and the release of IL-1β, IL-6, and TNF-α. SIRT1 knockdown reversed these effects, supporting SIRT1 as an intermediary between TBX21 and the WDR5–H3K4me3 axis. In the autologous blood-induced rat ICH model, TBX21 knockdown or SIRT1 overexpression restored the expression of occludin, claudin-3, and ZO-1 and reduced hematoma volume. These findings link suppression of the inflammatory axis to improved BBB-associated outcomes *in vivo*. However, the study did not directly test microglia-to-endothelial signaling (17). Beyond canonical histone modifications, emerging evidence has identified H3K18 lactylation (H3K18la) as a microglia-intrinsic epigenetic regulator after ICH. In a collagenase-induced murine model of ICH, H3K18la levels were markedly elevated in perihematomal microglia during the subacute phase. Inhibition of p300/CBP reduced H3K18la levels and aggravated white matter injury as well as long-term cognitive deficits. Microglial depletion likewise worsened outcomes, whereas the combination of microglial depletion and p300/CBP inhibition did not further exacerbate the injury. Collectively, these findings support the lactate–p300/CBP–H3K18la axis as a microglia-specific epigenetic mechanism that contributes to white matter repair and functional recovery after ICH (18). A Warburg-like shift toward glycolysis increases lactate production. Lactate can then serve as a substrate for p300/CBP-mediated histone lactylation, directly linking metabolic reprogramming to chromatin-based transcriptional regulation (41). In the ICH model, inhibition of p300/CBP reduced microglial H3K18la, impaired oligodendrocyte precursor cell recruitment, and aggravated white matter injury and long-term cognitive deficits (18). The lactate–p300/CBP–H3K18la axis therefore provides an epigenetic bridge linking glycolytic metabolism to repair-associated transcriptional regulation after ICH.

The opposing effects of histone lactylation do not represent a simple contradiction. In perihematomal microglia, p300/CBP-mediated H3K18la supports oligodendrocyte precursor cell recruitment, white matter repair, and long-term functional recovery during the subacute phase (18). In injured neurons, H3K14la suppresses PMCA2 expression, reduces calcium efflux, and promotes intracellular Ca²^+^ accumulation and ferroptosis during the early phase (42). The effect of histone lactylation therefore depends on the modified lysine, cell type, downstream transcriptional target, and injury stage. These findings also caution against broad inhibition of p300/CBP or histone lactylation. Such treatment may reduce harmful neuronal H3K14la but also disrupt protective microglial H3K18la. Cell- and residue-selective strategies are therefore needed after ICH.

The broader epigenetic programs governing microglial state transitions after ICH remain incompletely defined. Their effects are likely to vary across early inflammation, hematoma clearance, and tissue repair. Evidence from ischemic stroke and neurodegenerative models suggests that epigenetic mechanisms can influence the stability and reversibility of microglial states (39, 43). Direct validation in ICH is still needed.

### 3.3 Epigenetic regulation in neurons

Although glial cells represent major regulators of neuroinflammation after ICH, accumulating evidence indicates that neurons also undergo injury-induced epigenetic remodeling. These changes may contribute to both inflammatory amplification and increased neuronal vulnerability. Studies of neuronal apoptosis have shown that caspase- and calpain-dependent degradation of CBP/p300 reduces histone acetyltransferase activity and promotes histone hypoacetylation (44). This mechanism may be relevant to neuronal injury after ICH, although it has not been directly validated in hemorrhagic models. Notably, neuronal epigenetic responses after ICH may be spatially and temporally heterogeneous rather than uniformly repressive. A recent rat study found increased histone H3 and H4 acetylation in the bilateral sensorimotor cortices approximately 2 weeks after collagenase-induced ICH, together with changes in IGF-1 and BDNF expression (45). These tissue-level findings suggest that histone acetylation may participate in adaptive transcriptional responses during recovery. Neuronal epigenetic changes may alter stress-response and survival pathways after ICH. Direct evidence that these changes regulate the release of damage-associated signals is still lacking. HMGB1 contributes to neuronal injury after ICH, and its suppression reduces neuronal autophagy, apoptosis, and neurological deficits (46). A direct link between neuronal epigenetic remodeling and HMGB1 release has not been established.

More direct neuron-specific evidence has recently emerged from studies of histone lactylation. In both hemin-treated primary cortical neurons and mouse ICH models, H3K14 lactylation was increased in perihematomal neurons and promoted neuronal ferroptotic injury through transcriptional regulation of PMCA2-related calcium handling; inhibition of this pathway reduced neuronal degeneration and improved functional outcomes. These findings identify neuronal H3K14la as a distinct injury-promoting lactylation program after ICH (42). Neuron-resolved epigenomic studies after ICH remain limited. Future work should combine cell-type-specific profiling with causal perturbation to determine whether neuronal epigenetic changes actively contribute to inflammatory amplification or mainly reflect secondary injury.

### 3.4 Epigenetic regulation in endothelial cells

BBB failure after ICH follows a stage-dependent course. Within 6 h, endothelial swelling, shortened and poorly defined tight junctions, and BBB leakage are already detectable in both collagenase and autologous blood mouse models (47). The METTL3–YTHDF1–BCL-3 axis provides an m6A-dependent mechanism relevant to this early endothelial injury. METTL3-mediated m6A modification increases BCL-3 expression, while YTHDF1 supports BCL-3 translation. This pathway promotes lipid ROS and iron accumulation, apoptosis, and ferroptosis in brain microvascular endothelial cells after ICH (48). However, its activation within the first 6 h has not been directly defined.

Following the acute destructive phase, endothelial activation creates an immune-facilitation phase at 24 h after ICH. Levels of miR-126-3p are reduced in the hemorrhagic area after ICH. Restoring miR-126-3p lowers VCAM-1 expression and attenuates BBB disruption (49). A separate study linked its protective effect to the PIK3R2/Akt pathway. miR-126-3p replacement reduced BBB permeability, cerebral edema, and neutrophil infiltration (50). miR-27a-3p replacement also reduced vascular leakage, brain edema, and leukocyte infiltration at 24 h by suppressing endothelial AQP11 (51). Loss of these protective miRNA pathways may therefore favor leukocyte recruitment during acute BBB failure. Direct evidence that TET2 demethylates the ICAM-1 or VCAM-1 promoters after ICH is still lacking.

Between days 3 and 5, BBB damage reaches a model-dependent peak. Leakage and tight-junction injury are most severe on day 3 in the collagenase model and on day 5 in the autologous blood model (47). In a separate autologous blood ICH study, *Blnc1* was increased in the hematoma, perihematomal edema, and cerebral microvessels on day 3. *Blnc1* silencing reduced brain edema, BBB disruption, and inflammatory responses. The study linked these effects to the PPAR-γ/SIRT6/FOXO3 pathway (52).

From day 7 to day 14, BBB permeability declines but does not recover uniformly. In collagenase-induced rat ICH, ipsilateral permeability remained significantly elevated on day 7. By day 14, most leakage had resolved, although residual BBB dysfunction was still detected in 40% of animals (53). This interval therefore represents an incomplete restoration phase. The miR-126-3p–VCAM-1 and miR-27a-3p–AQP11 axes described above are potential counter-regulatory mechanisms for limiting barrier leakage and edema. Experimental restoration of miR-126-3p reduced VCAM-1 expression and BBB disruption (49). miR-27a-3p replacement suppressed AQP11 and reduced vascular leakage, leukocyte infiltration, and brain edema (51). However, these effects were produced by exogenous miRNA mimics during the acute phase. Endogenous upregulation of either pathway during subacute BBB repair has not been demonstrated.

One endothelial epigenetic pathway still cannot be assigned to a precise stage after ICH. *Snhg3* activates TWEAK/Fn14/STAT3 signaling, increases MMP-2/9 secretion, and promotes endothelial apoptosis and monolayer permeability. Its timing after ICH remains undefined (54).

Current evidence supports a sequence from hyperacute structural injury to 24-h immune facilitation, a model-dependent peak in barrier failure between days 3 and 5, and incomplete restoration from day 7 to day 14. This timeline is assembled from separate studies using different models and mostly single experimental endpoints. Endothelial-specific and time-resolved studies are still needed to define when these epigenetic pathways emerge, persist, and resolve during BBB repair.

### 3.5 Epigenetic regulation in infiltrating immune cells

Peripheral immune recruitment is an important component of neuroinflammation after ICH. The contribution of pre-existing and injury-induced epigenetic programs in infiltrating immune cells remains insufficiently defined in the ICH setting. Following blood–brain barrier disruption, neutrophils, inflammatory monocytes/macrophages, and lymphocyte subsets enter the perihematomal region, where they encounter a highly unusual inflammatory milieu enriched in hemoglobin degradation products, heme, iron, reactive oxygen species, cytokines, and DAMPs (5–7, 55). This environment is unlikely to simply “activate” incoming immune cells in a uniform manner. Rather, it may impose distinct transcriptional and epigenetic constraints that determine whether these cells amplify early tissue injury or participate in hematoma clearance and inflammatory resolution.

Infiltrating leukocytes enter the perihematomal region with pre-existing epigenetic programs shaped by lineage, age, prior inflammatory exposure, and clonal hematopoiesis. These cell-intrinsic states may interact with local hematoma-derived cues to influence inflammatory amplification, hematoma clearance, and resolution. However, direct cell-type-resolved studies of these interactions after ICH remain limited.

Evidence from broader studies of innate immunity and neuroinflammatory disease suggests that DNA methylation, histone modifications, and ncRNA networks can shape peripheral myeloid-cell inflammatory states, particularly inflammatory and repair-associated myeloid programs (56). These concepts are relevant to ICH, but they should not be assumed to operate identically in the hemorrhagic brain. Unlike many chronic neuroinflammatory disorders, ICH exposes infiltrating immune cells to an acute hematoma-derived stress niche characterized by iron overload, oxidative stress, and rapid erythrocyte breakdown. Such conditions may favor a distinct form of immune-cell reprogramming, potentially involving trained immunity-like myeloid chromatin remodeling during the injury-amplifying phase and epigenetic stabilization of reparative or regulatory programs during the resolution phase.

Another layer of peripheral immune priming may arise before immune cells enter the injured brain. Clonal hematopoiesis of indeterminate potential (CHIP) is an age-related condition commonly driven by somatic mutations in epigenetic regulators such as DNMT3A, TET2, and ASXL1 (57). These mutations can alter myeloid differentiation and inflammatory signaling, creating a cell-intrinsic bias toward heightened chemokine expression and inflammasome activation in monocytes and macrophages (58). Similar effects may influence circulating neutrophil responses, although the evidence is less direct. In ICH, CHIP may therefore modify the baseline state of infiltrating myeloid cells before they encounter hematoma-derived signals. CHIP has also been associated with a higher risk of stroke, including hemorrhagic stroke (59). Direct evidence linking CHIP to the epigenetic state or function of infiltrating immune cells after ICH remains limited. Nevertheless, this concept highlights the need to study peripheral epigenetic history alongside local perihematomal cues.

Future studies should move beyond documenting infiltrating immune cells in the perihematomal region. A key goal is to define how their epigenetic states differ from resident microglia and how these states evolve over time. Bulk perihematomal tissue analyses cannot resolve this question. Cell-type-resolved approaches, including single-cell transcriptomics, single-cell ATAC-seq, spatial profiling, and lineage-informed strategies, will be required to distinguish resident and recruited myeloid populations and to map epigenetic programs associated with injury amplification or inflammatory resolution after ICH. In this sense, infiltrating immune cells represent both a missing layer and a necessary test of the immuno-epigenetic framework proposed in this review.

## 4 Integrated immuno-epigenetic framework of neuroinflammation after ICH

Emerging evidence suggests that DNA methylation, histone modifications, and non-coding RNA–mediated regulation do not operate as independent modules after ICH. Instead, these mechanisms converge to form an integrated immuno-epigenetic regulatory network that shapes the trajectory of neuroinflammation after ICH.

Hematoma-derived DAMPs initiate innate immune activation and trigger rapid transcriptional responses in resident glial cells (3, 4). This response also alters chromatin regulation in resident glial cells. Activating histone marks can support inflammatory gene transcription (17). Time-resolved snATAC-seq has also revealed cell-type-specific changes in chromatin accessibility after ICH (9).

Inflammatory signaling can further reshape chromatin states by modulating the activity and recruitment of epigenetic writers and erasers, supporting a bidirectional interaction between immune activation and epigenetic remodeling (60). During the resolution phase, this inflammatory program may be partially restrained by repressive histone modifications and DNA remethylation.

Time-course epigenomic profiling further suggests that vascular-associated cell populations also undergo dynamic chromatin remodeling after ICH, although mechanistic resolution remains more limited than that available for glial programs (9).

Non-coding RNAs further integrate this network by fine-tuning the expression of epigenetic enzymes and inflammatory mediators (61). Through these interactions, DNA methylation, histone modifications, and RNA-based regulation converge to shape cell-type-specific inflammatory phenotypes and transitions between injury-amplifying and resolution-associated states. Thus, neuroinflammation after ICH should be conceptualized as an epigenetically encoded, cell-type-specific dynamic state rather than a purely cytokine-driven response.

### 4.1 Metabolic–epigenetic coupling in the perihematomal microenvironment

After ICH, erythrocyte lysis exposes the perihematomal tissue to hemoglobin degradation products, heme, free iron, and ROS. Iron-dependent oxidative reactions aggravate mitochondrial dysfunction and disrupt redox homeostasis (62–64). These metabolic changes may alter the availability of substrates and cofactors required by chromatin-modifying enzymes. This may contribute to epigenetic changes after ICH.

TET dioxygenases illustrate the complexity of this interaction. TET enzymes require Fe²^+^ and α-ketoglutarate to oxidize 5-methylcytosine, while ascorbate helps maintain the iron in its catalytically active state (65–67). In principle, local Fe²^+^ availability could influence TET catalysis. This does not mean that iron overload uniformly increases TET activity. Excess iron also promotes Fenton chemistry and ROS production (62, 63). In cerebral vascular endothelial cells, ROS reduced TET2-dependent 5hmC enrichment at the ZO-1 promoter (68). Whether the same mechanism occurs in perihematomal cells after ICH remains unknown. Changes in redox state and mitochondrial metabolism may also affect the conditions required for TET catalysis (66, 67). TET activity after ICH may therefore depend on the local balance among Fe²^+^ availability, redox state, and metabolic cofactor supply. Direct measurements of TET activity and 5-hydroxymethylcytosine dynamics in the perihematomal region remain limited. Current evidence does not support a uniformly hypermethylated or hypomethylated state after ICH. Global 5hmC is reduced in experimental ICH brain tissue, but this does not establish the direction of 5mC changes (69). Human whole-blood methylome studies have identified both hypermethylated and hypomethylated loci, with more hypermethylated sites among the significant changes (70). These findings come from different tissues and measure different aspects of DNA methylation. DNA methylation after ICH should therefore be interpreted according to tissue, cell type, genomic locus, and injury stage.

Other epigenetic pathways are also linked to cellular metabolism. S-adenosylmethionine (SAM) serves as the methyl donor for DNA and histone methyltransferases, and nicotinamide adenine dinucleotide (NAD^+^) is required for sirtuin deacetylase activity (71). Experimental ICH studies have shown that total NAD^+^ levels decrease early in perihematomal tissue. Nuclear and cell-type-specific NAD^+^ changes remain unclear (72). Direct evidence for altered SAM availability after ICH remains scarce. Acetyl-CoA, α-ketoglutarate, and redox cofactors provide additional links between cellular metabolism and histone acetylation or demethylation (73).

### 4.2 Endothelial cell–immune cell crosstalk

At the immune–vascular interface after ICH, endothelial epigenetic regulation links BBB injury to peripheral immune recruitment. Changes in endothelial survival, junctional integrity, and adhesion-related signaling alter barrier permeability and leukocyte entry (48–52, 54). Recruited immune cells then enter a vascular environment already shaped by hematoma-derived stress and inflammatory signaling. The endothelium forms a functional link between local vascular injury and the peripheral immune response.

### 4.3 Epigenetically relevant glial crosstalk after ICH

Compared with endothelial–immune interactions, direct evidence that epigenetic mechanisms govern glia–glia or glia–neuron crosstalk after ICH remains limited. Current evidence indicates extensive inflammatory communication among astrocytes, microglia, and neurons after ICH, but most of these findings involve cytokines, complement-related pathways, or inflammasome signaling rather than cell-type-specific epigenetic regulation. Activated microglia can transfer miR-383-3p to neurons through exosomes. Wei et al. showed that these vesicles delivered miR-383-3p into neurons. The transferred miRNA suppressed ATF4 and promoted neuronal necroptosis in cellular and rat ICH models (74). This mechanism allows an injury-related RNA signal to pass from microglia to nearby neurons.

Repeated exosome release and uptake may expose neurons beyond the immediate hematoma border to the same signal. This could contribute to the extension of secondary injury through perihematomal tissue. Spatial movement of these exosomes has not yet been tracked *in vivo*. The proposed spread away from the hematoma core therefore remains a possible mechanism.

## 5 Conclusions and future research

ICH is an acute vascular event, but its secondary injury phase also involves a dynamically evolving immuno-epigenetic process. Accumulating evidence indicates that epigenetic remodeling is more than a downstream consequence of injury. It can shape the intensity, duration, and cell-type specificity of neuroinflammation after ICH.

ICH induces coordinated, cell-specific epigenetic reprogramming across microglia, astrocytes, endothelial cells, neurons, and potentially infiltrating immune cells. DNA methylation shifts, histone mark redistribution, chromatin accessibility remodeling, and non-coding RNA networks may influence whether inflammatory responses resolve or persist over time. The dual-phase nature of neuroinflammation further suggests that epigenetic modifications may act as regulatory nodes that influence the balance between injury-amplifying and resolution-associated responses. Despite rapid progress, key challenges remain. The temporal trajectory of epigenetic remodeling after ICH is incompletely defined, and it remains unclear whether acute inflammatory signatures leave persistent “epigenetic scars” that influence long-term recovery. Moreover, most current insights derive from preclinical models, underscoring the need for cell-type–resolved and spatial epigenomic profiling in human ICH.

To date, direct epigenomic profiling (e.g., DNA methylation sequencing, ChIP-seq) of human ICH perihematomal brain tissue remains scarce. Although peripheral blood methylome studies have identified disease-associated CpG methylation signatures that distinguish ICH patients from healthy controls, these findings may not fully reflect the epigenetic state of the affected brain tissue (70). Consequently, comprehensive, brain tissue–specific epigenomic data remain critically lacking. Integrating perihematomal tissue from surgical procedures or post-mortem brain samples with genome-wide methylation and chromatin profiling (e.g., ChIP-seq, ATAC-seq) will be crucial for validating the conserved regulatory nodes identified in animal models and enhancing their translational relevance. Furthermore, persistent epigenetic alterations, including age-associated DNA methylation drift, have been consistently documented throughout the human lifespan. These alterations have been associated with sustained transcriptional dysregulation and progressive cellular dysfunction. Evidence from longitudinal and cross-sectional studies indicates that aging and chronic neuroinflammation are associated with DNA methylation drift, supporting its potential value as a biomarker of disease progression and clinical prognosis (75, 76). Epigenetic clocks based on DNA methylation profiles have shown prognostic potential in various diseases. Whether they can help predict long-term outcomes after ICH remains to be tested directly.

Future advances will likely depend on integrative multi-omics mapping and precision epigenetic interventions, including locus-specific epigenome editing and RNA-based therapeutics. The opposing effects of microglial H3K18la and neuronal H3K14la expose a major limitation of nonselective epigenetic therapy. Broad inhibition of p300/CBP or lactylation-related pathways could reduce neuronal H3K14la and ferroptosis while also disrupting microglial H3K18la-associated white matter repair. Cell-type-, residue-, and stage-selective targeting is therefore essential before clinical translation. Looking forward, CRISPR-dCas9 epigenome editors provide a practical way to test causal links between chromatin marks and inflammatory gene expression. dCas9-p300 can increase local histone acetylation, while dCas9-DNMT3A or dCas9-TET1 can modify DNA methylation at defined genomic regions. These experiments can then assess changes in gene expression and inflammatory phenotypes *in vitro* and *in vivo* (77, 78). Reframing ICH within an immuno-epigenetic paradigm may shift therapeutic strategies from broad anti-inflammatory suppression toward targeted molecular reprogramming, ultimately improving neurological recovery.
